# Statistical Validation of the Grand Rapids Arch Collapse Classification

**DOI:** 10.1177/2473011419834531

**Published:** 2019-05-10

**Authors:** David Burkard, Daniel Patton, Michelle Padley, John David Maskill, Donald Raymond Bohay, John Gregory Anderson

**Affiliations:** 1 Michigan State University College of Human Medicine, MI, USA; 2 Department of Orthopaedic Surgery, Arrowhead Regional Medical Center in Colton, CA, USA; 3 Orthopaedic Associates of Michigan in Grand Rapids, MI, USA

**Keywords:** flatfoot, posterior tibial tendon dysfunction, kappa, plantar fasciitis, ankle arthritis

## Abstract

**Background:**

The Grand Rapids Arch Collapse Classification system was devised in 2011 to assist physicians’ and patients’ understanding of the mechanisms underlying arch collapse. Five types of arch collapse are described, based on which part of the foot or ankle is affected. The purpose of this study was to determine the inter- and intrarater reliability of this classification system when used by physicians with various levels of training.

**Methods:**

A senior author identified a stratified selection of 50 patients (10 per classification type) who presented with foot/ankle pain and suitable radiographs. A survey was designed using prediagnosis radiographs and clinical synopses of the patient’s chart. The survey consisted of a description of the classification scheme and the 50 cases in a randomized order. Eight weeks later, they repeated the test to analyze for intra-rater agreement.

**Results:**

Of the 33 physicians who received the survey, 26 completed the first round (16 attendings, 4 foot and ankle fellows, and 6 residents). Overall, there was substantial agreement among raters in all five types. Kappa scores for each type were 0.72, 0.65, 0.72, 0.70, 0.63, respectively. The combined kappa score was 0.68. After 8 weeks, 13 of the 26 participants repeated the study. A Kappa analysis was once again performed for the 13 respondents, which produced a substantial level of agreement with a value of 0.74 for intrarater reliability.

**Conclusion:**

The Grand Rapids Arch Collapse Classification system was designed to provide an accessible mechanism for physicians to consistently describe arch collapse, its effects, and the conditions associated with it. The utility of this system is wholly reliant on the repeatability among clinicians. This study has demonstrated that the classification system has substantial rates of reliability among physicians of different levels of experience and training.

**Level of evidence:**

Level IV.

## Introduction

In 1989, Johnson and Strom proposed that posterior tibial tendon dysfunction led to degenerative arch collapse.^5,18^ In their model, there were 3 stages: stage 1 has no fixed deformity of the foot and ankle; stage 2 has a dynamic deformity of the hindfoot; finally, stage 3 has a fixed hindfoot deformity. Myerson later added a fourth stage to account for valgus tilt of the ankle.^23^ Since this model was developed back in 1989, foot and ankle surgeons have developed a better understanding of not only posterior tibial tendon dysfunction, but also the pathophysiology underlying many common conditions associated with arch collapse. With this deeper understanding, there comes a need for a more updated model to explain arch collapse and the conditions associated with it. The senior authors have published many studies delving into the conditions associated with arch collapse.^2,6,15,16,21,24^ The Grand Rapids Arch Collapse Classification System accounts for many common degenerative conditions associated with arch collapse.

The Grand Rapids Arch Collapse Classification (GRACC) was devised to describe the gradual progression of arch collapse due to tensile failure of the plantar apparatus of the foot. The classification system was designed around the premise that one of the major forces behind degenerative arch collapse is gastrocnemius contracture—defined as <10 degrees of ankle dorsiflexion with the knee in full extension.^2,8,14,20^

**Figure 1.**
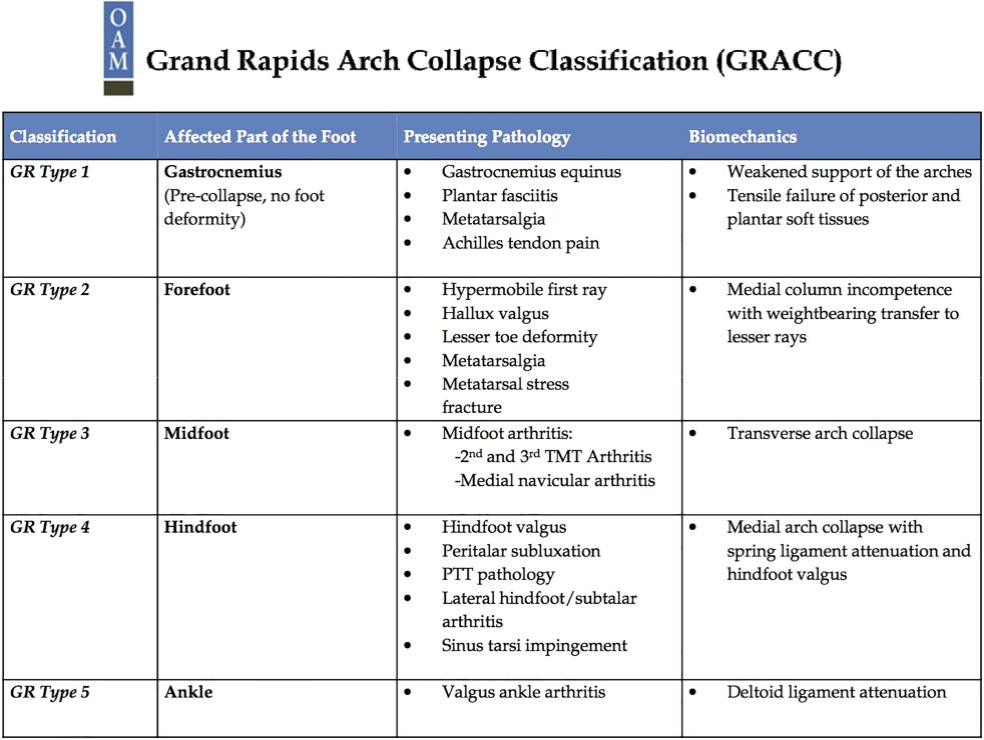
Grand Rapids Arch Collapse Classification overview, common presentations, and biomechanics.

Type I deformities result from gastrocnemius equinus; they are characterized by normal radiographs, including no arthritic changes, a normal talonavicular coverage angle, a normal midfoot linear relationship, and a normal sesamoid position.^10-13,28^ A type II deformity results from progressive medial column incompetence with weight bearing, leading to elevation of the first ray with an overload of the lesser metatarsal heads. These conditions can lead to the processes (Figure 1). They are characterized radiographically by normal navicular-cuneiform coverage with evidence of forefoot deformity and an elevated first ray.^3,4,8,17,30^ A type III deformity results from further dorsal compression leading to midfoot arthritis. On radiographs, type III deformities demonstrate second and third tarsometatarsal arthritis and medial dorsal navicular-cuneiform arthritis; however, talonavicular coverage remains normal.^21^ In type IV deformities, there is failure of both the posterior tibial tendon and/or spring ligament. Patients with type IV deformities typically present with hindfoot valgus, talar head uncovering, and subtalar joint subluxation or arthrosis. Ultimately, deltoid ligament attenuation can lead to type V deformity involving valgus tilting of the ankle and tibiotalar joint arthropathy.^25-27,30^

The authors hoped to develop an instrument that could be reliably used by physicians with varying experience. Its simplicity and potential for reliability make it an excellent resource for patient education as well as clinician decision making. In order for the GRACC to be considered a viable, useful classification system, it must be consistently reproducible by physicians. The primary goal of this study was to examine the inter- and intrarater reliability of the Grand Rapids Arch Collapse Classification.

## Materials and Methods

**Figure 2.**
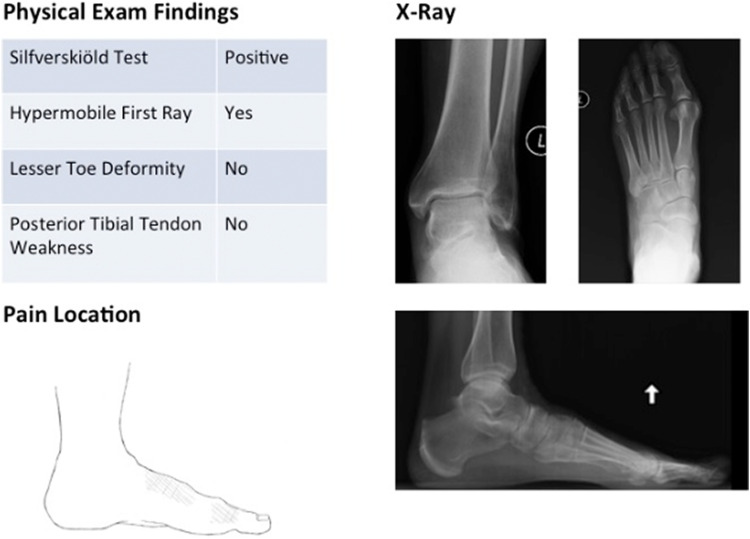
Example of physical exam findings, pain location, and radiographs as presented to each physician. (Illustration provided by Lindsey A. Behrend, BS)

**Figure 3.**
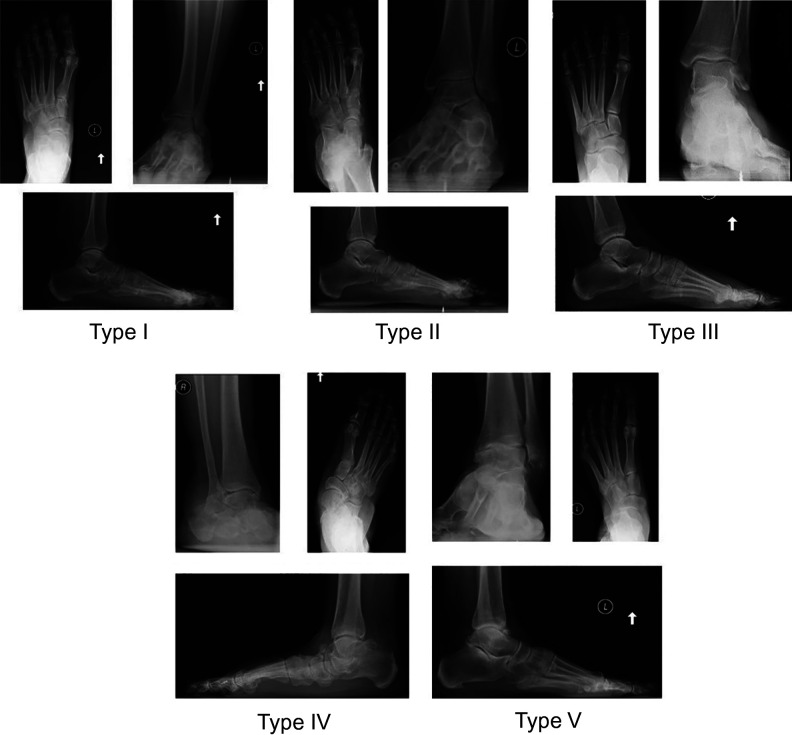
Radiographic examples of each of the five types of the Grand Rapids Arch Collapse Classification.

The study was determined to be exempt by the Spectrum Health IRB. An a priori power analysis determined that 50 clinical cases were required to achieve significant reliability among our cohort.^1,7,20^ Cases were randomly selected by a medical student who had no prior understanding of the classification system using CPT codes to filter patients. Most notably, 27687 for gastrocnemius recession was used to include patients who had undergone a gastrocnemius recession as this is involved with all 5 types of the GRACC. Charts were reviewed from 4 foot and ankle orthopedic surgeons from a single practice to identify 15 patients for each deformity type as documented in each patient’s encounter. The senior author then randomly selected 10 patients for each deformity type who had all necessary radiographs and physical exam findings documented. A survey was constructed using deidentified pretreatment radiographs and clinical synopses of corresponding patients (Figure 2). All subject radiographs included weightbearing anteroposterior and lateral foot as well as anteroposterior and lateral ankle views (Figure 3). Clinical synopses included pain location along with a list of relevant objective findings in the evaluation of foot and ankle pain. The clinical synopses included result of Silfverskiöld test, presence of a hypermobile first ray as determined by assessing motion at the first tarsometatarsal joint, presence of any lesser toe deformities, presence of posterior tibial tendon weakness. First ray hypermobility had been assessed by grasping and squeezing at the tarsometatarsal articulation and assessing the degree of movement with dorsal pressure to the first ray. PTT dysfunction was tested using a single-leg heel raise test. All tests had been performed by foot and ankle fellowship–trained orthopedic surgeons. Deidentified radiographs and chart summaries were presented in a randomized order. For each patient, they were asked to designate type I, II, III, IV, or V or Not Applicable if they thought the patient did not fit any of the designated types of deformity.

This test was sent out to 33 physicians of varying experience. Of the 33 physicians who received the test, 26 completed the first round (16 attending surgeons from various subspecialties, 4 foot and ankle fellows, and 6 senior orthopedic surgery residents) to evaluate interrater reliability. Of these 26 physicians, 13 completed the second round 8 weeks later in order to evaluate intrarater reliability.

An independent statistics group determined the reliability rating of each type of deformity for the GRACC using Cohen’s kappa coefficient analysis. The level of agreement for each kappa [κ] value was determined based on the recommendations by Cohen and Landis (Table 1).^9,19,22^ Reliability was determined both for inter- and intrarater observations for all 5 deformity types.

**Table 1.** Level of Agreement at Different [Kappa] Values as Recommended by Landis and Koch.

Kappa Value	Level of Agreement
0.81-1.00	Near perfect
0.61-0.80	Substantial
0.41-0.60	Moderate
0.21-0.40	Fair
0.00-0.20	Slight

## Results

The interobserver reliability average for all five deformity types showed substantial agreement with a κ value of 0.6839. The κ value for each deformity type was 0.7164, 0.6510, 0.7219, 0.7013, and 0.6291, respectively (Table 2). The intraobserver reliability demonstrated substantial agreement with a κ value of 0.744 (Table 3).

**Table 2.** Interrater Reliability by GRACC Deformity Type.

Deformity Type	Kappa Value
I	0.7164
II	0.6510
III	0.7219
IV	0.7013
V	0.6291
Average	0.6839

**Table 3.** Intraobserver Reliability for all 13 Physicians Who Completed the Test at Baseline and at 8 Weeks.

Subject	Kappa Value
1	0.713
2	0.899
3	0.696
4	0.890
5	0.696
6	0.772
7	0.619
8	0.525
9	0.874
10	0.739
11	0.778
12	0.609
13	0.860
Average	0.744

## Discussion

Results of this study showed substantial reliability among physicians of varying experience for interrater reliability. This reliability supports the authors’ hypothesis that the GRACC would be easy to adopt and implement. The consistency of agreement in this study demonstrates the consistent application that is crucial to the utility of any classification system. Furthermore, there was substantial intrarater reliability, indicating the ability of this classification to monitor a patient’s deformity progression over time. Future work can focus on measuring the validity and responsiveness of this classification scheme. In this case, validity would require comparing responses to gold standard responses. Responsiveness would be measured by observing how well this classification system works in patients whose deformity type changes over time.^29^

**Figure 4.**
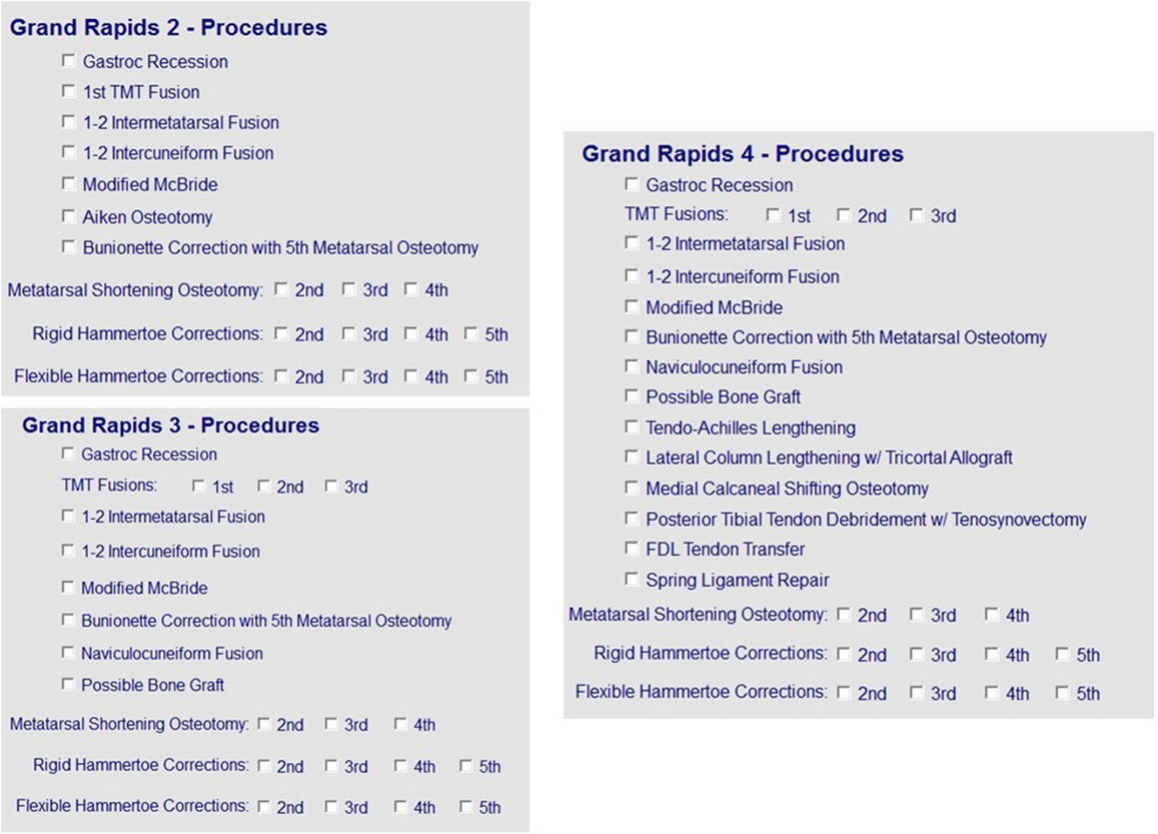
Examples of surgical templates used by a tertiary foot and ankle specialty clinic for over 18 years. These templates are used for patients classified with a type II, type III, and type IV deformities, respectively.

The surgical and nonsurgical treatment options depend on the arch collapse type. Patients with a type I deformity can be treated with physical therapy, orthotics, gastrocnemius recession, and/or tenoachilles lengthening. Type II deformities can be treated with physical therapy, orthotics, midfoot fusion, hammertoe correction, and/or shortening of long metatarsal bones. Type III deformities can be treated with physical therapy, orthotics, and/or midfoot joint fusions. Type IV deformities can be treated with orthotics, Arizona Brace, foot reconstruction with tendon transfers, subtalar joint fusion, and/or a triple fusion. Finally, type V deformities can be treated with orthotics, Arizona Brace, ankle joint replacement, and/or ankle joint fusion. Surgical templates have been created for each deformity type to streamline surgical decision making (Figure 4). Treatment with the above options for each deformity type has produced good outcomes.^2^

This study was limited by its relatively small sample size of physicians. Because of the low sample size, a greater number of patients than initially anticipated were required to achieve sufficient power. The large number of clinical vignettes that each physician had to work through could have led to fatigue by the end of the survey; randomization of the order of cases for each physician was used in an attempt to account for this bias. The study is further limited by its retrospective nature and any bias introduced by the creation of the clinical vignettes and image selection. These were minimized by having an inexperienced medical student extract the cases and using the same radiograph views for each patient. Recall bias was minimized for intrarater reliability by using a standardized 8-week delay in repeating the test. Finally, this study is limited by physical exam findings being given to the clinicians as opposed to evaluating themselves in the clinic. Ideally, clinicians would be able to independently evaluate each patient to obtain objective information; however, this was not possible in a study of this nature. Factors such as first ray mobility and, to a much lesser degree, gastrocnemius equinus can be somewhat subjective on clinical exam and likely represent a spectrum as opposed to a binary variable. However, for the purposes of this assessment, they were treated as binary variables to facilitate classification.

In summary, this study has demonstrated that the GRACC has substantial reliability among physicians with proper training on the classification system. This classification offers potential as a beneficial tool in guiding treatment and decision making for management of patients with adult acquired arch collapse.

## Supplemental Material

Supplemental Material, FAO834531-ICMJE - Statistical Validation of the Grand Rapids Arch Collapse Classification Click here for additional data file. Supplemental Material, FAO834531-ICMJE for Statistical Validation of the Grand Rapids Arch Collapse Classification by David Burkard, Daniel Patton, Michelle Padley, John David Maskill, Donald Raymond Bohay and John Gregory Anderson in Foot & Ankle Orthopaedics

## References

[1] Altman DG . Practical Statistics for Medical Research. London: Chapman and Hall, 1991.

[2] Anderson JG Bohay DR Eller EB Witt BL . Gastrocnemius recession. Foot Ankle Clin. 2014;19(4):767–786. 25456721 10.1016/j.fcl.2014.09.001

[3] Armstrong DG Stacpoole-Shea S Nguyen H Harkless LB . Lengthening of the Achilles tendon in diabetic patients who are at high risk for ulceration of the foot. J Bone Joint Surg. 1999;81:535–538. 10225799 10.2106/00004623-199904000-00011

[4] Aronow MS Diaz-Doran V Sullivan RJ Adams DJ . The effect of triceps surae contracture force on plantar foot pressure distribution. Foot Ankle Int. 2006;27(1):43–52. 16442028 10.1177/107110070602700108

[5] Beals TC Pomeroy GC Manoli A . Posterior tibial tendon insufficiency: diagnosis and treatment. J Am Acad Orthop Surg. 1999;7(2):112–118. 10336306 10.5435/00124635-199903000-00004

[6] Bohay DR Anderson JG Gentchos CE . Treatment of stage 2 posterior tibial tendon dysfunction. In: Coetzee JC Hurwitz SR , eds. Arthritis and Arthroplasty: The Foot and Ankle. Philadelphia, PA: Saunders; 2009:264–272.

[7] Bujang MA Baharum N . Guidelines of the minimum sample size requirements for Cohen’s Kappa. Epidemiol Biostat Public Health. 2017;14(2):e12267-1-10.

[8] Cheung JT Zhang M An K . Effect of Achilles tendon loading on plantar fascia tension in the standing foot. Clin Biomech (Bristol Avon). 2006;21:194–203. 10.1016/j.clinbiomech.2005.09.016 16288943

[9] Cohen J . A coefficient of agreement for nominal scales. Educ Psychol Measure. 1960;20:37–46.

[10] DiGiovanni CW Kuo R Tejwani N . Isolated gastrocnemius tightness. J Bone Joint Surg Am. 2002;84:962–970. 12063330 10.2106/00004623-200206000-00010

[bibr11-2473011419834531] DiGiovanni CW Langer P . The role of isolated gastrocnemius and combined Achilles contractures in the flatfoot. Foot Ankle Clin N Am. 2007;12:363–379. 10.1016/j.fcl.2007.03.005 17561207

[bibr12-2473011419834531] Downey MS Banks AS . Gastrocnemius recession in the treatment of nonspastic ankle equinus; a retrospective study. J Am Podiatr Med Assoc. 1989;79:159–174. 2732918 10.7547/87507315-79-4-159

[13] Duthon VB Lubbeke A Duc Sylvain SR . Noninsertional Achilles tendinopathy treated with gastrocnemius lengthening. Foot Ankle Int. 2011;32(4):375–379. 21733439 10.3113/FAI.2011.0375

[14] Geng X Wang C Ma X . Mobility of the first metatarsal-cuneiform joint in patients with and without hallux valgus: in vivo three-dimensional analysis using computerized tomography scan. J Orthop Surg Res. 2015;10:140. 26370272 10.1186/s13018-015-0289-2 PMC4570606

[15] Gentchos CE Anderson JG Bohay DR . Management of the rigid arthritic flatfoot in the adults: alternatives to triple arthrodesis. Foot Ankle Clin N Am. 2012;17:323–335. 10.1016/j.fcl.2012.03.009 22541529

[16] Habbu R Holthusen SM Anderson JG Bohay DR . Operative correction of arch collapse with forefoot deformity: a retrospective analysis of outcomes. Foot Ankle Int. 2011;32(8):764–773. 22049862 10.3113/FAI.2011.0764

[17] Hill RS. Ankle equinus: Prevalence and linkage to common foot pathology. J Am Podiatr Med Assoc. 1995;85:295–300. 7602500 10.7547/87507315-85-6-295

[18] Johnson KA Strom DE . Tibialis posterior tendon dysfunction. Clin Orthop Relat Res. 1989;239:196–206. 2912622

[19] Landis JR Koch GG . The measurement of observer agreement for categorical data. Biometrics. 1977;33:159–174. 843571

[20] Mackinnon A . A spreadsheet for the calculation of comprehensive statistics for the assessment of diagnostic tests and inter-rater agreement. Comput Biol Med. 2000;30(3):127–134. 10758228 10.1016/s0010-4825(00)00006-8

[21] Maskill JD Bohay DR Anderson JG . Gastrocnemius recession to treat isolated foot pain. Foot Ankle Int. 2010;31(1):19–23. 20067718 10.3113/FAI.2010.0019

[22] McHugh ML . Interrater reliability: the kappa statistic. Biochem Me (Zagreb). 2012;22(3):276–282. PMC3900052 23092060

[23] Myerson MS . Adult acquired flatfoot deformity: Treatment of dysfunction of the posterior tibial tendon. Instr Course Lect. 1007;46:393–405. 9143981

[24] Nemec SA Habbu RA Anderson JG Bohay DR . Outcomes following midfoot arthrodesis for primary arthritis. Foot Ankle Int. 2011;32(4):355–361. 21733436 10.3113/FAI.2011.0355

[25] Pinney S Lin S . Current concept review: acquired adult flatfoot deformity. Foot Ankle Int. 2006;27(1):66–73. 16442033 10.1177/107110070602700113

[bibr26-2473011419834531] Pomeroy GC Pike H Beals T Manoli A . Acquired flatfoot in adults due to dysfunction of the posterior tibial tendon. J Bone Joint Surgery. 1999;81:1173–1182. 10.2106/00004623-199908000-00014 10466651

[27] Popovic N Lemaire R . Acquired flatfoot deformity secondary to dysfunction of the tibialis posterior tendon. Acta Orthop Belg. 2003;69:211–222. 12879702

[28] Sammarco GJ Mahesh RB Sammarco VJ . The effects of unilateral gastrocnemius recession. Foot Ankle Int. 2006;27(7):508–511. 16842717 10.1177/107110070602700705

[29] Tarrant C Angell E Baker R . Responsiveness of primary care services: development of a patient-report measure—qualitative study and initial quantitative pilot testing. Southampton (UK): NIHR Journals Library (Health Services and Delivery Research, No. 2.46). Chapter 3, Literature review: meaning and measurement of responsiveness, 2014. 25642526

[30] Van Boerum DH Sangeorzan BJ . Biomechanics and pathophysiology of flat foot. Foot Ankle Clin. 2003;8(3):419–430. 14560896 10.1016/s1083-7515(03)00084-6

